# Tasmanian devil cathelicidins exhibit anticancer activity against Devil Facial Tumour Disease (DFTD) cells

**DOI:** 10.1038/s41598-023-39901-0

**Published:** 2023-08-04

**Authors:** Cleopatra Petrohilos, Amanda Patchett, Carolyn J. Hogg, Katherine Belov, Emma Peel

**Affiliations:** 1 School of Life and Environmental Sciences, The University of Sydney, Sydney, NSW Australia; 2 Australian Research Council Centre of Excellence for Innovations in Peptide & Protein Science, The University of Sydney, Sydney, NSW Australia; 3 Menzies Institute for Medical Research, University of Tasmania, Hobart, TAS Australia

**Keywords:** Cancer genetics, Immunogenetics

## Abstract

The Tasmanian devil (*Sarcophilus harrisii*) is endangered due to the spread of Devil Facial Tumour Disease (DFTD), a contagious cancer with no current treatment options. Here we test whether seven recently characterized Tasmanian devil cathelicidins are involved in cancer regulation. We measured DFTD cell viability in vitro following incubation with each of the seven peptides and describe the effect of each on gene expression in treated cells. Four cathelicidins (Saha-CATH3, 4, 5 and 6) were toxic to DFTD cells and caused general signs of cellular stress. The most toxic peptide (Saha-CATH5) also suppressed the ERBB and YAP1/TAZ signaling pathways, both of which have been identified as important drivers of cancer proliferation. Three cathelicidins induced inflammatory pathways in DFTD cells that may potentially recruit immune cells in vivo. This study suggests that devil cathelicidins have some anti-cancer and inflammatory functions and should be explored further to determine whether they have potential as treatment leads.

## Introduction

The Tasmanian devil (*Sarcophilus harrisii*) is the largest extant marsupial carnivore^1^, playing a vital role in maintaining the diversity and resilience of the Tasmanian ecosystem^2–4^. The species is currently listed as Endangered due to Devil Facial Tumour Disease (DFTD)^5^, a contagious cancer that is spread by biting during social interactions^6^. DFTD was first observed in 1996 in NE Tasmania^7^, however in 2012 a second transmissible cancer was found, giving rise to the naming convention of DFT1 for the first form of DFTD, and DFT2 for the more recent form^8^. All further mentions of DFTD in this paper refer to DFT1. Much has been written on Tasmanian devils, their immune systems, DFTD and the interaction between devils and the disease (see^9^ for a comprehensive review).

Currently, there are no treatment options for DFTD although there are research efforts into developing a vaccine. Histocompatibility barriers between different major histocompatibility complex class I (MHC-I) molecules ordinarily prevent the proliferation of allograft tissue^10^. However, as DFTD cells do not express MHC-I molecules, they are able to evade recognition by the host immune system^11^.Vaccine candidates have focused on upregulating expression of MHC-I in DFTD cells using interferon gamma^12^. Although these have successfully induced anti-DFTD antibodies within vaccinated individuals^12^, this has not been sufficient to prevent infection long term^13,14^. However, unlike unvaccinated devils, the tumours in vaccinated animals exhibited immune cell infiltration^14^ and regressed with immunotherapy^13^.

Other drug candidates have been identified as potential treatments for DFTD and show promising anticancer activity in vitro but have not yet progressed to clinical trials*.* DFTD cells are sensitive to Receptor Tyrosine Kinase (RTK) inhibitors in vitro, particularly Afatinib^15^. This inhibits the ErbB2 receptor, resulting in suppression of the ErbB3-STAT3 axis. The ErbB3-STAT3 axis influences genes involved in the cell cycle and angiogenesis and its overexpression is linked to cancer metastasis^16^. It also enables DFTD cells to evade the host immune system by downregulating expression of MHC-I molecules^17^. Imiquimod, a guanosine analogue, similarly suppresses elements of the ErbB3-STAT3 axis, as well as inducing DFTD cell apoptosis via oxidative stress and the unfolded protein response (UPR)^18^. Recently, statins such as Atorvastatin have also shown therapeutic potential against DTFD^19^. Atorvostatin disrupts cholesterol homeostasis which is an important driver of DFTD. In addition, Atorvastatin prevented DFTD cell proliferation in vitro with no effect on Tasmanian devil fibroblasts, and induced necrosis of DFTD xenografts in nude mice^19^.

We propose that a novel treatment for DFTD may be derived from cathelicidins, a major class of vertebrate antimicrobial peptides. Cathelicidins are small cationic peptides that share a conserved N-terminal preprosequence^20^. They have pleiotropic properties that extend beyond direct antimicrobial activity, including chemotaxis, wound healing and angiogenesis^21^. Cathelicidins also regulate cancer in a highly tissue specific manner: in some types of tumours they are overexpressed and promote tumorigenesis^22–26^, while in others they exhibit anticancer properties^27–29^. For example, cathelicidin-BF (BF-30), a peptide from the venom of the banded krait (*Bungarus fasciatus*), suppresses proliferation of the murine melanoma cell line B16F10 by interfering with the transcription of vasoendothelial growth factor (VEGF)^30^. Similarly, bovine myeloid antimicrobial peptide 28 (BMAP‑28) suppresses the proliferation of human thyroid cancer TT cells in xenograft murine models^31^, and the caprine cathelicidin ChMAP-28 shows selective cytotoxicity against multiple human cell lines in vitro via necrotic mechanisms^32^. Multiple peptides derived from the porcine cathelicidin tritrpticin exhibit selective cytotoxicity towards Jurkat T cell leukemia line in vitro via a membranolytic mechanism^33^.

Cathelicidins have been isolated from many vertebrate taxa^34–37^. However, they have undergone gene expansion in marsupials due to the need for additional protection of immunologically naïve young during development in the pouch^38^. Unlike eutherians, marsupials are born immunologically naïve and do not develop a mature immune response until many months postpartum^39^. During development within the pouch, young are exposed to diverse microbial flora, some of which are pathogenic^38^. This has likely encouraged the lineage-specific expansion of cathelicidins in marsupials, resulting in multiple diverse peptides: 36 cathelicidins have been identified in four species to date^40–43^, including 7 in the Tasmanian devil^44^. Many marsupial cathelicidins have exhibited potent antimicrobial activity against a range of microorganisms in vitro while being non-toxic to mammalian cell lines up to concentrations of 500 µg/mL^40,44,45^. Of the three devil cathelicidins with antimicrobial activity (Saha-CATH3, 5 and 6), Saha-CATH3 is not haemolytic to human red blood cells while the other two are moderately haemolytic are high concentrations above 250 μg/mL^44^. However, their anticancer potential has not yet been explored. Here, we investigate the cytotoxicity of seven Tasmanian devil cathelicidins against the DFT1 cell line 1426 in vitro*,* and explore potential mechanisms of action using RNAseq, with the aim to identify peptide candidates for future development as anti-DFTD therapeutics.

## Materials and methods

### Peptide preparation and cell lines

Tasmanian devil cathelicidin mature peptides Saha-CATH1, 2, 3, 4, 5, 6 and 7^44^ (Supplementary Table 1) were synthesized by ChinaPeptides Co. Ltd to 95% purity by high performance liquid chromatograph (HPLC). All peptides were solubilized in water for cell culture (Sigma-Aldrich) with 0.01% glacial acetic acid at a concentration of 10 mg/mL, then serially diluted twofold in Roswell Park Memorial Institute (RPMI) 1640 media to give a final concentration of 1 mg/mL to 31.25 μg/mL.

The DFT1 cell line 1426, derived from a primary DFT1 tumour^46^, was obtained from Menzies Institute for Medical Research, University of Tasmania. The cell line was maintained in RPMI 1640 media with 2 mM l-glutamine (Gibco) supplemented with 10% AmnioMax II (Gibco), 10% heat-inactivated foetal bovine serum (FBS) and 1% penicillin/streptomycin (Sigma-Aldrich), in T75 flasks (Nunc) at 35 °C 5% CO_2_. Cells were passaged using trypsin–EDTA (Sigma-Aldrich) when 90% confluent.

### Cytotoxicity assay

Toxicity of seven Tasmanian devil cathelicidins against DFT1 1426 cells was determined for twofold dilutions from 500 to 15.62 µg/mL, and four different timepoints (12, 18, 24 and 36 h). Confluent DFT1 1426 cells (passage 22) were re-suspended in RPMI 1640 with 10% FBS and 10% AmnioMax II at a concentration of 5 × 10^5^ cells/mL, and 100μL seeded into each well of a sterile flat-bottom 96 well plate (Corning), with individual plates for each timepoint (12, 18, 24 and 36 h). All plates were incubated for 24 h at 35 °C 5% CO_2_ prior to the cytotoxicity test.

At 0 h, 100 μL of each peptide dilution was then added to each plate in quadruplicate for all timepoints (12, 18, 24 and 36 h), resulting in a final peptide concentrations of 500 μg/mL, 250 μg/mL, 125 μg/mL, 62.50 μg/mL, 31.25 μg/mL and 15.62 μg/mL. To ensure that the solvent did not affect absorbance values, a vehicle control was also included for the maximum incubation period (36 h). An untreated growth control of RPMI 1640, a positive control of dimethylsulfoxide (DMSO) (15%) and sterility control of RPMI 1640 only (no cells) were also included for each time point.

For the 12 h timepoint, 10% alamarBlue was immediately added to each well of the plate after addition of the peptide. For the remaining timepoints, plates were incubated for an additional 6, 12 or 24 h after addition of the peptides, then 10% alamarBlue added to each well. For all timepoints, after the addition of alamarBlue cells were incubated for a further 12 h, resulting in a total peptide incubation period of 12, 18, 24 and 36 h. Absorbance of alamarBlue for all timepoints was determined at 570 nm and 630 nm on a Biotek 800 TS microplate reader. Cell viability was calculated according to manufacturer’s instructions and expressed as a percentage of cell survival compared to the untreated growth control.

A one-sample, one-tailed t-test was used to test for significant difference in viability between treated cells and the negative growth control. A one sample, two-tailed t-test was also conducted to ensure there was no significant difference in viability between the negative growth control and the cells treated with the solvent only. Statistical analysis was conducted in R (R Development Core Team 2021).

### Mechanism of action

RNAseq was used to characterize the mechanisms underlying the anticancer activity of the peptides. Confluent DFT1 1426 cells (passage number 25) were re-suspended at a concentration of 5 × 10^5^ cells/ml and 1 mL seeded into each well of a sterile flat-bottom polystyrene 12-well plate (Corning). Following incubation for 24 h at 35 °C 5% CO_2_, media was aspirated from the cells and replaced with 500 μL of RPMI 1640 with 2 mM l-glutamine, 10% FBS and 10% AmnioMax II. 500 μL of each 1 mg/mL peptide solution (Saha-CATH1 to 7) was then added to the plate in triplicate, to give a final peptide concentration of 500 μg/mL. This was the highest concentration tested in the previous cytotoxicity assay and hence would likely lead to maximal peptide activity, enabling precise analysis of changes in gene expression. A vehicle control was also added to the plate in triplicate.

The plate was then incubated for a further 10 h at 35 °C in 5% CO_2_. This incubation period was selected as the cytotoxicity assay indicated it was sublethal for most of the peptides and would maintain sufficient cell viability to extract intact RNA. Media was then aspirated from the cells, which were washed twice with Dulbecco’s phosphate buffered saline (DPBS) (Sigma-Aldrich).

Total RNA was extracted from each replicate of the peptide-treated and vehicle control cells (n = 3) using the RNeasy mini kit (Qiagen) with cell lysis directly in each well of the 12 well plate. Total RNA was quality assessed using the RNA nano 6000 kit on the Agilent Bioanalyzer with all samples displaying a RIN score between 5 and 9. In total, 24 RNA samples corresponding to three treatments per peptide (Saha-CATH1 to 7) and a vehicle control were submitted to Ramaciotti Centre for Genomics (The University of New South Wales) for sequencing. Illumina TruSeq mRNA libraries were prepared for all samples, which were sequenced as 2 × 150 bp paired-end reads across an SP flowcell on the NovaSeq6000. This resulted in 27 to 50 million raw reads per sample.

Raw reads were quality assessed using FastQC v0.11.8^47^, then quality and length trimmed using Trimmomatic v0.39^48^ using default parameters. Trimmed reads for each treatment (n = 3) and the control (n = 3) were aligned to the Tasmanian devil reference genome v7.0 (NCBI: GCA_000189315.1^49^ using STAR v2.7.8a^50^. Alignments for each treatment were summarized into gene counts using featureCounts in the subread package v1.5.1^51^.

Gene counts were used as input for differential expression analysis in R^52^. Genes with less than 50 counts across all samples were removed from the analysis, as these were unlikely to be biologically relevant and lacked sufficient statistical power. Firstly, the data was normalized by trimmed mean of M values (TMM) using edgeR v3.32.1^53^ to account for any composition bias between libraries and to give an effective library size for downstream analysis. Multidimensional scaling (MDS) was used to check for variation between treatments using limma v3.36.0^54^. Expression levels were then normalized using upper-quartile normalization in EDAseq v2.24.0^55^ to account for differences in distribution between lanes, such as sequencing depth. Differential expression analysis was performed using voom in the limma v3.36.0 package^54^.

For each treatment, a false discovery rate (FDR) cutoff of 0.02 was applied and genes that were up or downregulated greater than 1.5× fold were selected for Gene Ontology (GO) and Ingenuity Pathway Analysis (IPA)^56^. Over-representation analysis of Biological Processes was conducted in clusterProfiler v3.18.1^57^. Statistical significance was adjusted for multiple comparisons using the Benjamini–Hochberg method, and terms were considered significant when p-adj < 0.05. To remove general terms, gene sets larger than 200 were removed, and the simplify function was used to remove redundant GO terms. Further pathway analysis was conducted in IPA (Qiagen)^58^, using mammal as the species type and nervous system for the tissue/cell type.

## Results

**Figure 1:**
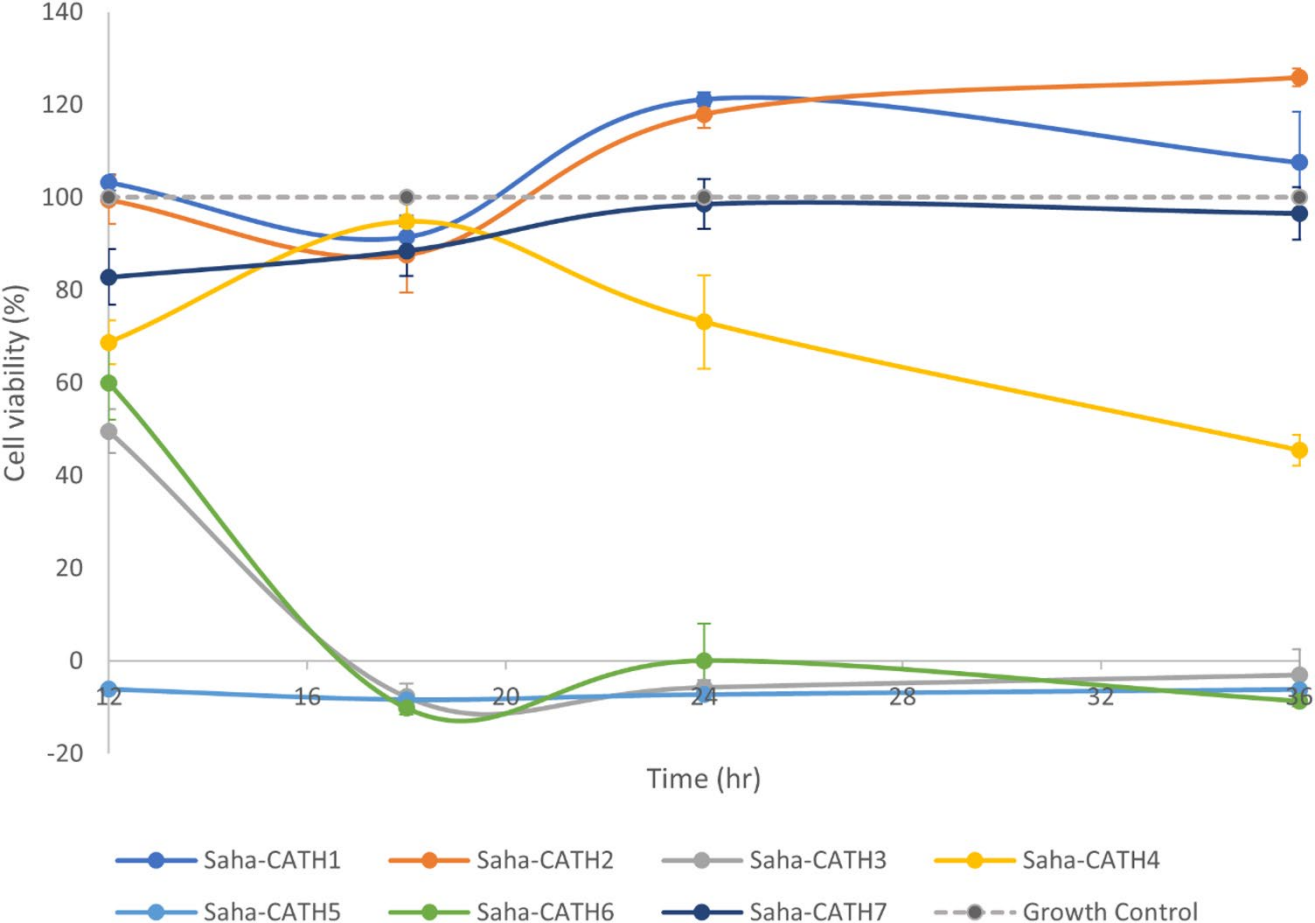
Changes in DFT1 1426 cell viability over time at peptide concentrations of 500 µg/mL. Cell viability is expressed as a percentage of cell survival compared to the untreated growth control. The mean values ± SD (error bars) of the assay performed in quadruplicate are reported. 3 of the toxic peptides (Saha-CATH3, Saha-CATH5 and Saha-CATH6) reduced cell viability to 0% after 18 h, while Saha-CATH4 reduced cell viability by 50% after 36 h. The other 3 the peptides (Saha-CATH1, Saha-CATH2 and Saha-CATH7) did not reduce cell viability.

Four Tasmanian devil cathelicidins (Saha-CATH3, 4, 5 and 6) significantly decreased cell viability by more than 50% over 36 h compared to the growth control at 500 μg/mL. Saha-CATH5 displayed the most rapid cytotoxic activity against DFT1 cells, and reduced cell viability to less than 0% at all time points (p-value < 3.63E−09) (Fig. 1). This negative viability is likely caused by the change in media pH over the incubation period. Cell viability was calculated by measuring the amount of reduced alamarBlue (AR). This calculation requires a correction factor to allow for the oxidized substrate present in the media. In cases of very low survival, the change in pH between the media of the treated cells and the media used to calculate the correction factor can result in a negative AR value. Saha-CATH3 (p-value < 2.44E−06) and Saha-CATH6 (p-value < 7.12E−05) required an 18-h incubation period before exhibiting a similar level of toxicity to Saha-CATH5. Saha-CATH4 also significantly reduced cell viability (p-value = 0.04) but was slower acting, requiring 36 h to induce significant cytotoxicity by more than 50%.

Saha-CATH3, 5 and 6 also reduced cell viability by more than 50% at lower concentrations (Supplementary Fig. 1). Saha-CATH5 was cytotoxic at concentrations ≥ 125 µg/mL (p-value < 5.56E−04) at all time points, while Saha-CATH6 was cytotoxic at concentrations $\ge$ 250 µg/mL at incubation periods ≥ 18 h (p-value < 1.05 E−04). Saha-CATH3 was cytotoxic at concentrations between 62.5 and 250 µg/mL at incubation periods ≥ 18 h (p-value < 1.12 E−04). The exception was the Saha-CATH3 concentration of 62.5 µg/mL at 24 h (mean cell 360 viability 78.8% ± 21.4sd), although this result was not statistically significant (p = 0.07).

Differential expression (DE) analysis identified 12,402 DE genes out of a total of 15,548 across all seven treatments when compared to the control. Most of these were in the Saha-CATH5 treatment (11, 514 or 74.05%). The other toxic treatments had between 10 and 20% DE genes—Saha-CATH3 had 1, 963 (12.63%), Saha-CATH4 had 2, 915 (18.75%) and Saha-CATH6 had 2, 419 (15.56%). All the DFT1 cells treated with the non-toxic peptides Saha-CATH1, 2 and 7 had less than 1% DE genes, with Saha-CATH7 having 0 under the quality filters chosen. Due to the low number of differentially expressed genes, these treatments were excluded from further analysis.

**Figure 2:**
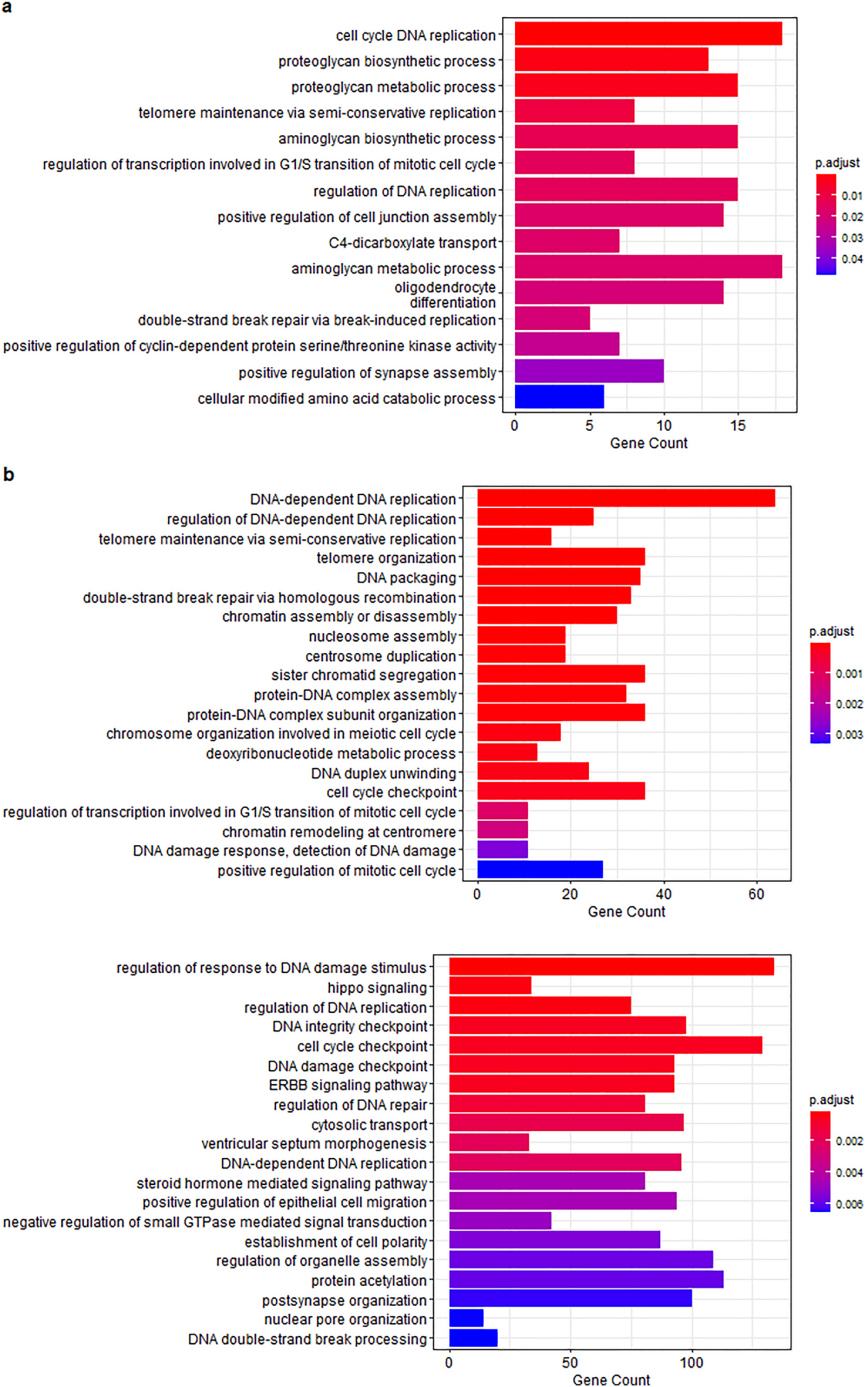
GO terms downregulated in (**a**) SahaCATH3 treatment, (**b**) SahaCATH4 treatment and (**c**) SahaCATH5 treatment. Terms associated with cell cycle and DNA repair/checkpoints were downregulated in all three. In Saha-CATH5 treatment, terms associated with ERBB and YAP1 signalling were also downregulated.

Treatment of DFT1 cells with Saha-CATH3, 4 and 5 resulted in downregulation of genes involved in DNA replication, cell cycle progression and checkpoints (Table 1). This was supported by both GO (Fig. 2) and IPA analysis (Supplementary Table 2). Canonical pathways significantly inhibited by treatment with these peptides included ‘cell cycle control of chromosomal replication’ (Saha-CATH4: p-value = 9.64E−06; Saha-CATH5: p-value = 1.85E−02) and ‘cyclins and cell cycle regulation’ (Saha-CATH3: p-value = 3.27E−02; Saha-CATH4: p-value = 1.22E−02).

**Table 1:** DE genes in SahaCATH3, 4 and 5 treatments involved in DNA replication and cell cycle progression.

Function	Gene	Cathelicidin	Fold change	False discovery rate
G1-S Phase transition	*CCND1*	Saha-CATH5	0.52	2.54E−15
G1-S Phase transition	*CCND3*	Saha-CATH3	0.62	9.16E−10
G1-S Phase transition	*CCND3*	Saha-CATH4	0.35	6.30E−16
G1-S Phase transition	*CCNE1*	Saha-CATH3	0.57	1.67E−08
G1-S Phase transition	*CCNE1*	Saha-CATH4	0.44	1.98E−11
G1-S Phase transition	*CCNE1*	Saha-CATH5	0.37	5.17E−15
G1-S Phase transition	*CCNE2*	Saha-CATH3	0.61	7.83E−09
G1-S Phase transition	*CCNE2*	Saha-CATH4	0.31	4.61E−15
G1-S Phase transition	*CCNE2*	Saha-CATH5	0.30	7.93E−18
Cyclin transcription factor	*E2F2*	Saha-CATH3	0.48	9.28E−18
Cyclin transcription factor	*E2F2*	Saha-CATH4	0.28	3.29E−22
Pre-replication complex	*ORC1*	Saha-CATH4	0.46	2.28E−11
Pre-replication complex	*ORC1*	Saha-CATH5	0.54	1.14E−11
Pre-replication complex	*CDC6*	Saha-CATH4	0.29	8.76E−16
Pre-replication complex	*CDC6*	Saha-CATH5	0.39	1.65E−16
Pre-replication complex	*CDC7*	Saha-CATH4	0.38	3.99E−20
Pre-replication complex	*CDC45*	Saha-CATH5	0.42	6.51E−16

**Table 2:** Genes involved in immune cell activation upregulated in Saha-CATH3, 4 and 6 treatments.

Function	Gene	Cathelicidin	Fold change	False discovery rate
Cytokine	*IL6*	Saha-CATH3	4.38	0.0019
Cytokine	*IL6*	Saha-CATH4	3.73	0.0050
Cytokine	*IL6*	Saha-CATH6	11.14	1.02E-06
Cytokine receptor	*IL6R*	Saha-CATH3	2.88	4.56E-06
Cytokine receptor	*IL6R*	Saha-CATH4	5.25	3.62E-10
Cytokine receptor	*IL6R*	Saha-CATH6	1.70	0.012
Immune signaling	*MYD88*	Saha-CATH3	2.64	4.57E-11
Immune signaling	*MYD88*	Saha-CATH4	4.15	2.01E-15
Immune signaling	*MYD88*	Saha-CATH6	1.78	9.77E-07
Transcription factor	*RUNX1*	Saha-CATH3	2.71	2.58E-07
Transcription factor	*RUNX1*	Saha-CATH4	2.43	1.46E-06
Transcription factor	*RUNX1*	Saha-CATH6	1.61	0.0046
Cytokine receptor	*IL1R1*	Saha-CATH3	2.41	0.00031
Cytokine receptor	*IL1R1*	Saha-CATH6	2.29	0.00045
Cytokine receptor	*IL21R*	Saha-CATH6	1.58	0.0019

Alongside cell cycle arrest, Saha-CATH5 also regulated the ERBB and Hippo signaling pathways in DFT1 cells based on GO analysis (Fig. 2). IPA also revealed these canonical pathways were enriched in the treatment (‘Hippo signaling’: p-value = 1.64E−07; ‘ERBB’: p-value = 9.14E−04; ‘ERBB2-ERBB3’: p-value = 1.34E−03; ‘ERBB4’: p-value = 1.43E−04) (Supplementary Table 2). The ERBB3 gene was significantly downregulated by Saha-CATH5 (FC = 0.34, FDR = 9.99E−22).

**Figure 3:**
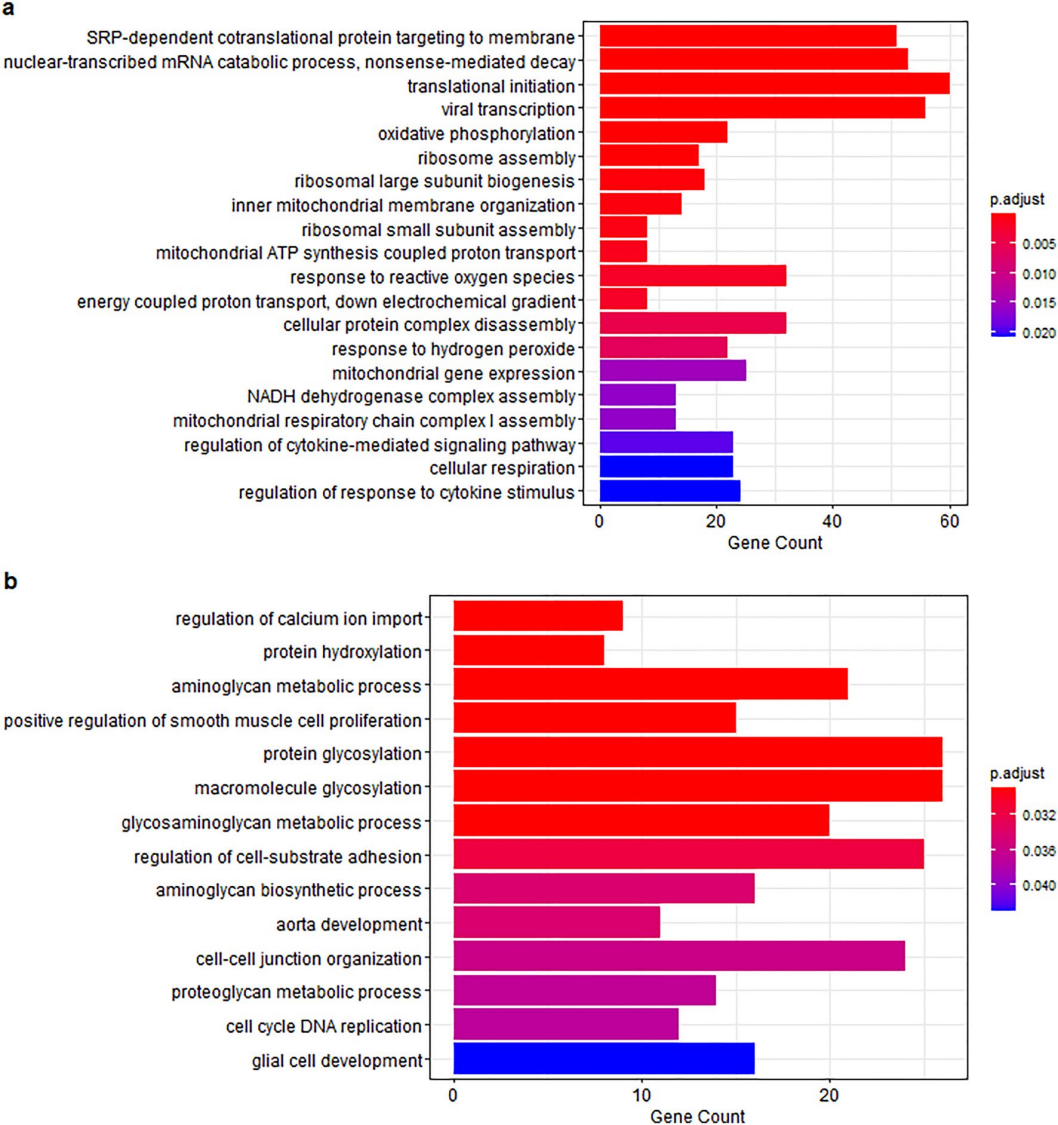
GO terms (**a**) upregulated and (**b**) downregulated in Saha-CATH6 treatment. Terms associated with an immune response were upregulated. Treatment also indicated signs of ER stress.

Saha-CATH6 induced Endoplasmic Reticulum (ER) stress in DFT1 cells via multiple mechanisms according to GO analysis (Fig. 3). IPA results confirmed some elements of ER stress were enriched in the Saha-CATH6 treatment. The most significant canonical pathway that was enriched was ‘EIF2 signaling’ (p-value = 1.33E−11), with GADD34 (PPP1R15A: FC = 2.14, FDR = 2.15E−19), CHOP (DDIT3: FC = 1.91, FDR = 1.03E−06) and ATF3 (FC = 5.02, FDR = 2.28E−16) all upregulated (Supplementary Table 2).

Interestingly, Saha-CATH3, 4 and 6 upregulated multiple genes involved in cytokine expression and immune signaling (Table 2). GO analysis further supported this, with multiple immune processes upregulated in the three treatments, including angiogenesis, wound healing, immune cell differentiation and cytokine response (Fig. 3a, Supplementary Fig. 2). IPA analysis indicated that the canonical pathway ‘Th17 activation’ was significantly enriched by Saha-CATH3 (p-value = 6.55E−05), Saha-CATH4 (p-value = 3.17E−03) and Saha-CATH6 (p-value = 2.55E−03) (Supplementary Table 2).

## Discussion

Here we aimed to identify cathelicidin candidates for future development as anti-DFTD therapeutics. Four Tasmanian devil cathelicidins (Saha-CATH3, 4, 5 and 6) were toxic to DFT1 1426 cells at high concentrations: Saha-CATH3 at concentrations ≥ 62.5 µg/mL; Saha-CATH4 at 500 µg/mL; Saha-CATH5 at concentrations ≥ 125 µg/mL and Saha-CATH6 at concentrations ≥ 250 µg/mL. Previous studies have shown that two of these peptides (Saha-CATH3 and 4) are non-toxic to human cell line A549^44^. The other two (Saha-CATH5 and 6) reduced cell viability at high concentrations (500 µg/mL), resulting in 42% and 59% cell survival respectively^44^. At the same concentrations and incubation period in this experiment, Saha-CATH5 and 6 resulted in 0% cell survival (Fig. 1). Interestingly, the fastest acting peptide has also shown broad- spectrum antimicrobial activity^44^. One of the toxic peptides (Saha-CATH4) has not previously shown antimicrobial activity in vitro. This may indicate that Saha-CATH4 also has antimicrobial activity against pathogens that have not been tested or is slower acting against the strains it has been tested against.

Saha-CATH3, 4 and 5 induced cell cycle arrest at the G1/S phase. The cell cycle is composed of four phases (M, G1, S, G2) during which DNA is replicated and the cell is divided in two^59^. Cell progression from one phase to the next is primarily triggered by cyclin dependent kinases (CDKs). Although CDKs are constantly expressed, they only become activated upon binding with a specific cyclin subunit^60^. Cyclin expression oscillates throughout the four phases to coordinate the cell cycle^61^. The transition between the G1 and S phase is triggered by cyclins D and E and signifies the point at which cell growth ceases and DNA replication begins^62,63^. It is accompanied by an increase in proteins that form the pre-replication complex (pre-RC) required for chromosomal replication such as ORC1^64^. Both the cyclins and elements of the pre-RC are overexpressed in tumours and have been identified as potential therapeutic targets^65–71^.

Many of these elements were downregulated in DFT1 1426 cells treated with Saha-CATH3, 4 and 5 (Table 1). This aligns with the activity of cathelicidins from other species that also suppress genes involved in DNA replication and cell cycle progression^72,73^. The data suggests that Saha-CATH3, 4 and 5 were inducing cell cycle arrest at the G1/S phase and should be further explored as potential therapeutics.

Alongside cell cycle arrest, Saha-CATH5 also regulated the ERBB and Hippo signaling pathways. It is particularly interesting that the ERBB3 gene was significantly downregulated by Saha-CATH5. This receptor has undergone copy gains in the DFTD genome^15^ and the ERBB-STAT3 axis has been identified as an important driver of DFTD^17^. Although DFTD cells are sensitive to multiple RTK inhibitors, they appear to respond most to those acting via this pathway^15^. This suggests Saha-CATH5 may be acting via a similar mechanism.

The oncogenic activity of RTK signaling pathways is often amplified by the formation of a positive feedback loop with the Hippo pathway^74,75^. When the Hippo pathway is dysregulated, the transcription factor YAP1 accumulates in the nucleus^76,77^. This can cause overexpression of many genes involved in proliferation and survival^78^. WWC3 attenuates this process by phosphorylating YAP1 to prevent its nuclear translocation^79^. WWC3 has undergone hemizygous deletion in DFTD, potentially resulting in overexpression of YAP1^15^. Therefore, the high toxicity of Saha-CATH5 against DFT1 1426 cells may be due to the peptide targeting the synergistic activity of both RTK signalling and YAP1 expression.

RTK inhibition has not yet been documented for any cathelicidins. The human cathelicidin LL-37 has even shown the opposite by activating two RTKs (EGFR and ERBB2), promoting tumour progression in certain cell lines^23,26^. However, RTK inhibitors are a major class of therapeutics against a wide variety of cancers^80,81^. This reveals an important property of Saha-CATH5 that has strong potential for future drug development as a therapeutic for DFTD.

GO analysis of Saha-CATH6 treatment suggested ER stress. These include glycosylation inhibition, calcium depletion and elevated reactive oxygen species (ROS)^82–85^. ER stress is induced when excessive misfolded proteins start to accumulate and disrupt homeostasis^84^. The unfolded protein response (UPR) aims to alleviate this stress by reducing protein translation, increasing chaperone expression, and degrading malformed proteins. If prolonged, the UPR leads to apoptosis^86^.

Other enriched GO terms in Saha-CATH6-treated DFT1 cells also indicated signs of ER stress. For example, protein hydroxylation was downregulated, suggesting attenuation of ER function that is characteristic of a stress response. Increased mRNA catabolism may be indicative of Regulated Ire1-Dependent Decay (RIDD). This process is upregulated during the UPR and involves degrading mRNAs to reduce the burden on the ER^87^. GO terms also suggested that translation was increased. Although this may appear contradictory as global translation is reduced during ER stress, the expression of ER chaperones and ERAD components is upregulated^85^.

This is one of three main pathways activated by the UPR. It attenuates global translation, while increasing expression of protective proteins^88–90^. The other two main pathways involved in the UPR are activated by inositol-requiring enzyme 1-a (IRE1a) and activating transcription factor 6 (ATF6)^86^. The increased mRNA catabolism in this treatment suggests RIDD is occurring, which may indicate activation of IRE1a. There is no evidence that the third pathway (ATF6) was enriched. However, the RNA from this treatment had degraded prior to sequencing (RIN = 5) which may influence the results. We recommend further studies using a lower concentration of Saha-CATH6 to confirm this mechanism of action.

GO and IPA results suggests a cytokine milieu within Saha-CATH3, 4 and 6-treated DFT1 cells that encourages Th17 differentiation, mirroring the activity of other vertebrate cathelicidins^91^. Pro-inflammatory cytokines can play a paradoxical role in cancer progression. Studies have indicated that cathelicidin-induced inflammation can promote the development of certain types of tumours^24^. However, many of these genes have also been shown to contribute to antitumour activity. There is evidence of interleukin-1 and -6 enhancing the cytotoxic activity of immune cells against cancer^92–94^. The transcription factor RUNX1 regulates expression of genes that inhibit leukaemia and breast cancer cells^95,96^. Further research is required to determine which of these dual roles predominate in DFTD.

**Figure 4:**
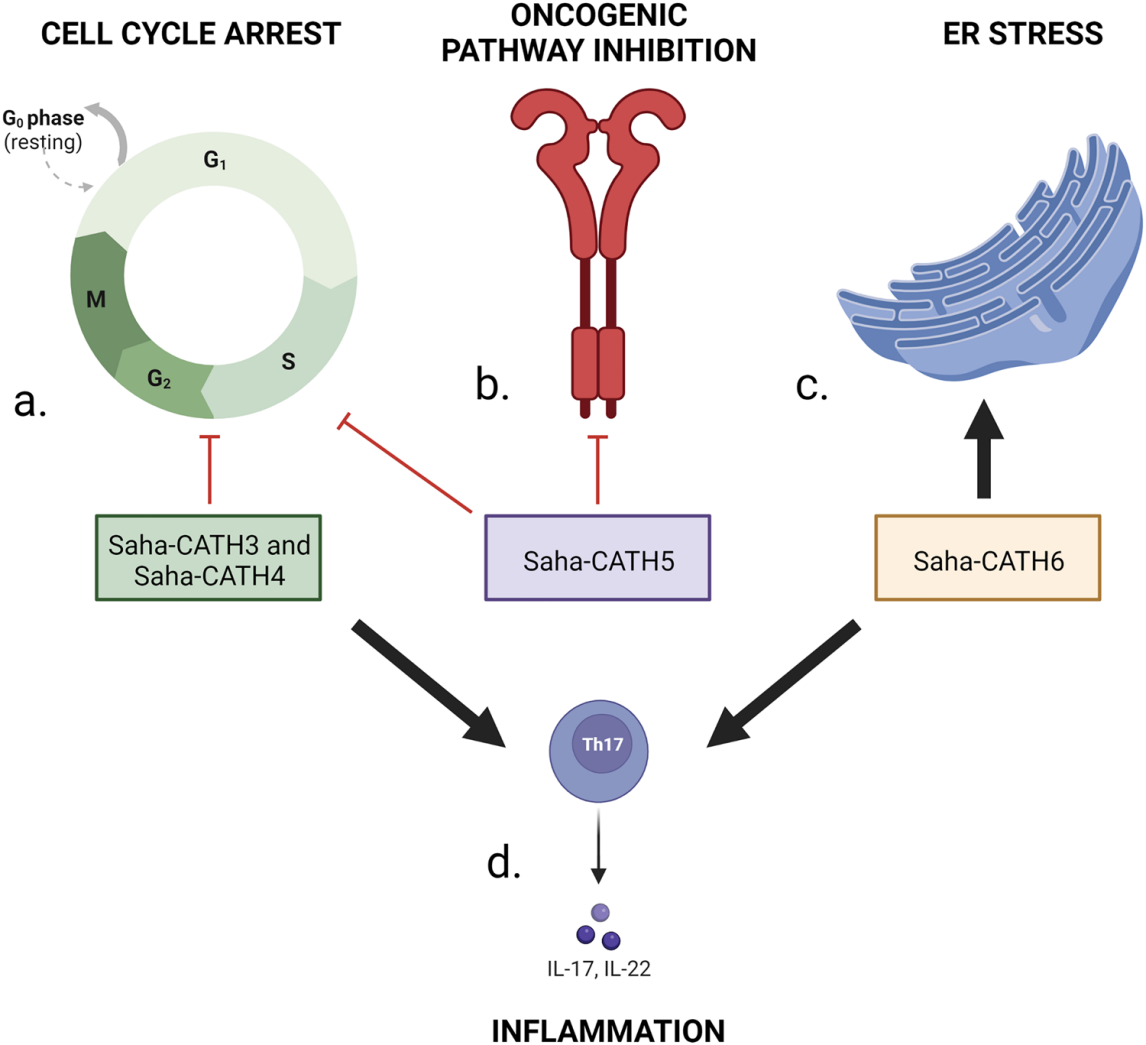
(**a**) Saha-CATH3, 4 and 5 induced cell cycle arrest at the G1/S phase; (**b**) Saha-CATH5 inhibited RTK signalling pathways and YAP1 expression; (**c**) Saha-CATH6 caused ER stress; (**d**) Saha-CATH3, 4 and 6 activated inflammatory cytokines. Figure created with BioRender.com.

In this study, we have identified four Tasmanian devil cathelicidins (Saha-CATH3, 4, 5 and 6) with cytotoxic activity against DFT1 cells in vitro. Using RNAseq we show that this activity is mediated via peptide-specific mechanisms (Fig. 4). Some of these mechanisms mirror the activity of other therapeutics currently being trialled against DFTD cells. For example, Saha-CATH5 inhibited the ERBB-STAT3 axis similarly to Afatinib^15^ and imiquimod^18^. Our results also reveal that three devil cathelicidins (Saha-CATH 3, 4 and 6) may induce inflammatory pathways in DFT1 cells involving increase cytokine expression. We suggest that future studies validate the pathway analyses using qRT-PCR. As the peptides have not been tested on healthy devil cells, we recommend that future studies investigate what effect cathelicidins have on fibroblasts. We also suggest that the peptides are tested against other human cancer cell lines to elucidate their broader anti-cancer functions. This will establish the selectivity of the peptides’ toxicity and determine whether they have future potential for drug development.

### Supplementary Information

Supplementary Information 1.

Supplementary Information 2.

## Data Availability

The raw sequencing reads generated and analyzed during this study are available in the National Centre for Biotechnology Information (NCBI) short read archive under BioProject number PRJNA970848.
