# Supplementary material for: Tasmanian devil cathelicidins exhibit anticancer activity against Devil Facial Tumour Disease (DFTD) cells
Source: Sci Rep. 2023 Aug 4;13:12698. doi: 10.1038/s41598-023-39901-0 (PMC10403513; doi:10.1038/s41598-023-39901-0)
Supplement: Supplementary file 1 — Supplementary Information 1. [file 41598_2023_39901_MOESM1_ESM.docx]

**SUPPLEMENTARY MATERIALS**

Table S1. Cathelicidin sequences and properties

| Cathelicidin name | Mature Peptide sequence | Length | MW | Charge at pH7 | GRAVY score | % positively charged AA | Amphipathic? |
| --- | --- | --- | --- | --- | --- | --- | --- |
| SahaCath1 | GIKHILFMAKTKLPRATCTAEIKENCDRKK | 30 | 3445.15 | 5.1 | -0.583 | 26.67 | Yes |
| SahaCath2 | TFKRKNGSRKNGHRPGGYSLIALGNKKVLKAPYMESI | 37 | 4117.81 | 8.1 | -0.868 | 24.32 | Yes |
| SahaCath3 | KRMGIFHLFWAGLRKLGNLIKNKIQQGIENFLG | 33 | 3841.62 | 5.1 | -0.079 | 18.18 | Yes |
| SahaCath4 | KREDFLDQIIRDFRNFIYQKYRRLRDEFRKLRDILSG | 37 | 4820.52 | 3.9 | -1.138 | 29.73 | Yes |
| SahaCath5 | KRIGLGGLIGRILDRLRRLG | 20 | 2232.75 | 6.9 | 0.03 | 30.00 | Yes |
| SahaCath6 | KRIRFFERIRDRLRDLGNRIKNRIRDFFS | 29 | 3794.43 | 6.9 | -1.162 | 37.93 | No - Hydrophilic |
| SahaCath7 | STILEDPGKIRERRDIGEEPIQKKQKMYRDQMQ | 33 | 4017.56 | 0.9 | -1.615 | 24.24 | No - Hydrophilic |

Table S2. Ingenuity Pathway Analysis Results

| **Treatment** | **Ingenuity Canonical Pathways** | **-log(p-value)** | **Ratio** | **z-score** | **Molecules** |
| --- | --- | --- | --- | --- | --- |
| Saha-CATH3 | Cyclins and Cell Cycle Regulation | 1.49E00 | 1.74E-01 | -2.3333333333333335 | ATM,CCND3,CCNE1,CCNE2,E2F2,E2F7,E2F8,HDAC7,HDAC9,PPP2R2B,SKP2,TGFB1 |
| Saha-CATH4 | Cyclins and Cell Cycle Regulation | 1.91E00 | 2.46E-01 | -3.317 | CCND3,CCNE1,CCNE2,CDKN1A,E2F2,E2F7,E2F8,HDAC2,HDAC7,HDAC9,PA2G4,PPP2R5B,RBL1,SKP2,TFDP1,TGFB1,TGFB2 |
| Saha-CATH4 | cell cycle control of chromosomal replication | 5.02E00 | 4.25E-01 | -3.638 | CDC45,CDC6,CDC7,CDK10,CDK18,CHEK2,DNA2,MCM4,MCM7,MCM8,ORC1,PCNA,POLA2,PRIM1,PRIM2,RPA2,TOP2A |
| Saha-CATH5 | cell cycle control of chromosomal replication | 1.73E00 | 7E-01 | -3.780 | CDC45,CDC6,CDC7,CDK1,CDK10,CDK13,CDK14,CDK17,CDK19,CDK5,CDK8,CDK9,CHEK2,DNA2,MCM8,MCM9,ORC1,ORC2,ORC3,ORC4,ORC5,PCNA,POLA2,PRIM1,RPA1,RPA3,TOP2A,TOP2B |
| Saha-CATH5 | Hippo signalling | 6.79E00 | 8.19E-01 | 0.898 | AMOT,BTRC,CD44,CRB1,CSNK1D,CSNK1E,CUL1,DLG1,DLG3,DLG4,DLG5,FAT4,FRMD6,ITCH,LATS1,LATS2,LLGL1,MOB1A,PATJ,PPM1L,PPP1CA,PPP1CB,PPP1R10,PPP1R12A,PPP1R14B,PPP1R14C,PPP1R3C,PPP1R3D,PPP1R7,PPP2CA,PPP2CB,PPP2R1B,PPP2R2A,PPP2R2B,PPP2R2C,PPP2R5B,PPP2R5E,SAV1,SCRIB,SKP1,SKP2,SMAD1,SMAD2,SMAD3,SMAD4,SMAD5,STK4,TEAD1,TEAD3,TJP2,TP53BP2,WWC1,WWTR1,YAP1,YWHAB,YWHAG,YWHAH,YWHAQ,YWHAZ |
| Saha-CATH5 | ERBB signaling | 3.04E00 | 6.99E-01 | -6.068 | AKT1,CDC42,EGF,ERBB2,ERBB3,ERBB4,FOS,FOXO1,GRB2,GSK3B,HBEGF,KRAS,MAP2K1,MAP2K2,MAP2K3,MAP2K4,MAPK10,MAPK11,MAPK12,MAPK14,MAPK8,MAPK9,MRAS,NCK2,NRAS,NRG1,NRG2,PAK2,PAK5,PDPK1,PIK3C2A,PIK3C3,PIK3CA,PIK3CB,PIK3R1,PIK3R2,PIK3R3,PRKCA,PRKCB,PRKCD,PRKCE,PRKCG,PRKCH,PRKCI,PRKD3,RALA,RALB,RAP1A,RAP1B,RAP2A,RAP2B,RASD1,RPS6KB1,RRAS,SHC1,SOS1,SOS2,TGFA |
| Saha-CATH5 | ERBB2-ERBB3 | 2.87E00 | 7.36E-01 | -4.439 | AKT1,BAD,CCND1,CDKN1B,ERBB2,ERBB3,ETV4,FOXO1,GRB2,GSK3B,KRAS,MAP2K1,MAP2K2,MRAS,NRAS,NRG1,NRG2,PDPK1,PIK3C2A,PIK3C3,PIK3CA,PIK3CB,PIK3R1,PIK3R2,PIK3R3,PTEN,RALA,RALB,RAP1A,RAP1B,RAP2A,RAP2B,RASD1,RRAS,SHC1,SOS1,SOS2,SP1,STAT3 |
| Saha-CATH5 | ERBB4 signaling | 3.84E00 | 7.63E-01 | -5.425 | ADAM17,AKT1,APH1A,APH1B,ERBB4,GRB2,KRAS,MAP2K1,MAP2K2,MRAS,NCSTN,NRAS,NRG1,NRG2,PDPK1,PIK3C2A,PIK3C3,PIK3CA,PIK3CB,PIK3R1,PIK3R2,PIK3R3,PRKCA,PRKCB,PRKCD,PRKCE,PRKCG,PRKCH,PRKCI,PRKD3,PSEN1,PSEN2,PSENEN,RALA,RALB,RAP1A,RAP1B,RAP2A,RAP2B,RASD1,RRAS,SHC1,SOS1,SOS2,YAP1 |
| Saha-CATH6 | EIF2 signaling | 1.09E01 | 2.98E-01 | 4.352 | AKT1,ATF3,DDIT3,EIF2B3,EIF3G,EIF4E,FAU,PIK3R3,PPP1CA,PPP1R15A,RPL10,RPL11,RPL12,RPL13,RPL13A,RPL17,RPL18,RPL21,RPL22,RPL23,RPL23A,RPL26,RPL27,RPL27A,RPL28,RPL30,RPL31,RPL32,RPL35,RPL36,RPL36AL,RPL37A,RPL38,RPLP0,RPLP2,RPS10,RPS14,RPS15,RPS15A,RPS16,RPS17,RPS21,RPS26,RPS27,RPS27A,RPS29,RPS5,RPS7,RRAS,VEGFA |
| Saha-CATH3 | Th17 activation pathway | 4.18E00 | 2.94E-01 | 3.3166247903554 | CD247,IL12RB1,IL1R1,IL6,IL6R,IRAK2,MYD88,NFAT5,NFATC2,NFKB1,REL,RELB,RUNX1,SOCS3,STAT3 |
| Saha-CATH4 | Th17 activation pathway | 2.5E00 | 2.94E-01 | 3.3166247903554 | CD247,IL12RB1,IL6,IL6R,IRAK2,IRAK4,MYD88,NFATC2,NFATC4,NFKB1,REL,RELB,RUNX1,SOCS3,STAT3 |
| Saha-CATH6 | Th17 activation pathway | 2.59E00 | 2.55E-01 | 2.5298221281347035 | CD247,FOXP3,IL12RB1,IL1R1,IL21R,IL6,IL6R,IRAK2,MYD88,NFATC2,RELB,RUNX1,SOCS3 |


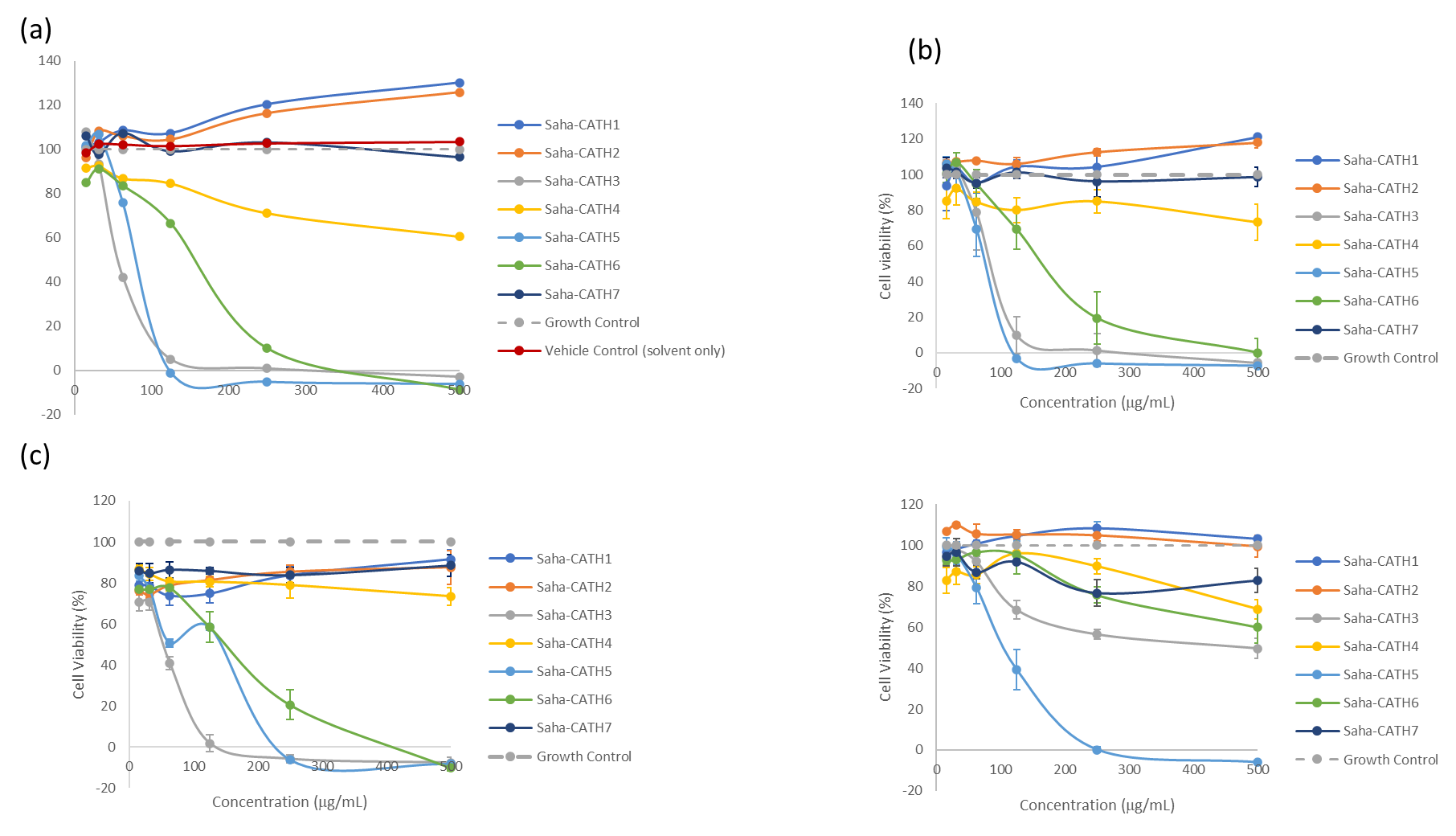


Figure S1: Changes in DFT1 1426 cell viability at different concentrations at (a) 36 hour incubation (b) 24 hour incubation (c) 12 hour incubation (d) 18 hour incubation. Cell viability is expressed as a percentage of cell survival compared to the untreated growth control. The mean values ± SD (error bars) of the assay performed in quadruplicate are reported. The vehicle control (solvent only) was used in the maximum incubation period to ensure that it did not impact cell growth at the concentrations used in the experiment.


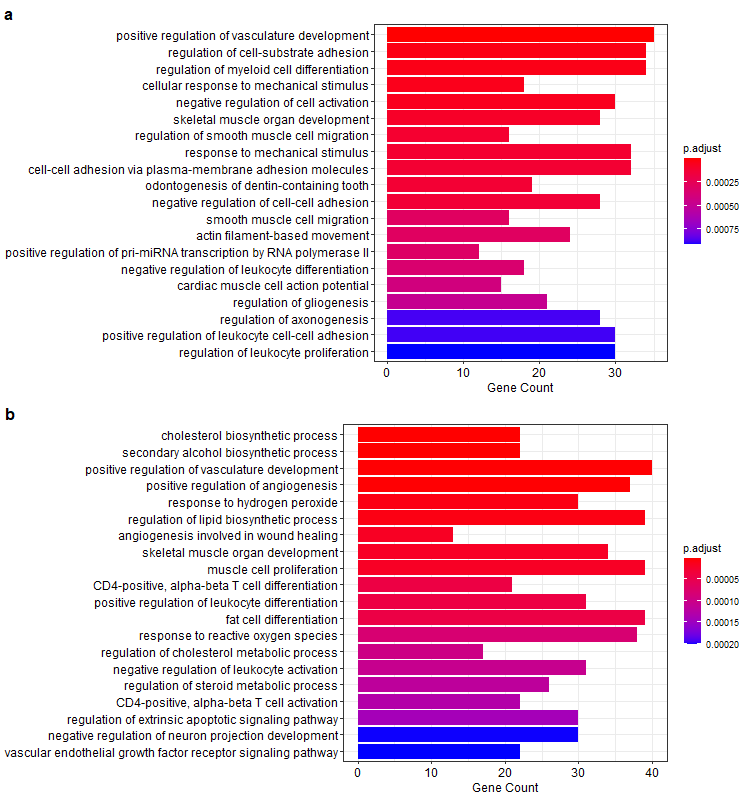


Figure S2: Upregulated GO terms associated with (a) Saha-CATH3 treatment and (b) Saha-CATH4 treatment. These indicated activation of an immune response.
